# The College of Nursing Badge

**Published:** 1920-04-24

**Authors:** 


### THE MATRONS' AND SISTERS' DEPARTMENT.

### THE COLLEGE OF NURSING BADGE.

The handsome badge of the College of Nursing, with its monogram N.C. on royal blue enamel will be soon becoming familiar on many uniforms. Its value to members is unique. It testifies to the wearer's admittance to the largest and most highly qualified professional body of nurses in this or any other country. For a considerable number of years the College badge will reckon as an immeasurably higher distinction than that of the Central Registration Board. No nurse who is able to satisfy the demands of the College Register can afford to dispense with this hall-mark of efficiency. She may possess the badge of her training-school, and we note that the custom of giving badges to qualified nurses is gaining ground in large training-centres, but if she lack that of the College she will run the risk of finding her training badge unrecognised when she passes out of the area within which it is sacred. The College Badge will become before very long a passport all over the world among professional nurses. It will open the gates of confidence and friendship in places where trained nurses are pioneers, and form a link between qualified members of a profession which includes at the present moment many irregularly-trained and even untrained workers.

Now although these are incontrovertible facts, although the badge of the Registration Board cannot during the next fifteen or twenty years carry the same weight of testimony to training as that of the College, there is one important sphere of action into which registration alone can introduce the nurse. Registration is, for the moment, not unlike the Giant painted by Dante in the Inferno, with monstrous arms hanging idle by his sides. No one can quite foresee what use will be made of the limbs which are at present untaught. The first Nursing Council has yet to be nominated by the Minister of Health. On this will rest the onus of setting up machinery and getting the untried wheels and cogs into motion. But its functions will not continue for very long. In the course of two, perhaps three, years, the nominated Nursing Council will retire and give place to one elected by registered nurses. Unless the fully-qualified members of the profession accept registration an extraordinary anomaly will result. The partially trained women at present practising as nurses will control the election of the Nursing Council by their votes, and a subtle lowering of the standard can hardly fail to result.

Every one anticipates that there will be a great rush of *bonâ fide* nurses to place themselves on the State Register when the lists are opened. There is a deplorably large number of nurses in almost every branch of the profession whose qualifications are shaky in some particular according to modern notions of efficiency. All these birds, black, white and grey, will come flocking to roost under the spreading branches of State registration. It does not by any means follow that they will find there the refuge they seek. The black will almost certainly be eliminated in the preliminary stage of enquiry. The grey, who have an unfortunate talent for putting forth black feathers as time goes on, can be eliminated under the penal clauses which will operate to keep the profession pure. The white none would wish to see excluded. But every one ought to appreciate the importance of not leaving the whole course of future nursing legislation to be settled by the votes of just the very persons whom it is desired in the future to reform out of existence.

Both badges, therefore, that of the College of Nursing and that of the Registration Board, are essential for nurses who aim at the consolidation of the profession. The College badge is necessary because it guarantees that the wearer is no mere *bonâ fide* nurse, but has been able to comply with some of the strictest regulations as regards training ever likely to be framed by public authority. The Registration Badge is necessary because it satisfies State requirements and confers on its wearer not merely the status of the registered, of which we had something to say lately, but the right to control by vote vital matters of nursing education and policy. To hold stiffly aloof from registration on the ground that the distinction it confers is not good enough for the College member since it has to be shared with professional inferiors is to surrender the right to take part in the great efforts still necessary to ensure peaceable and consistent progress. To abstain from getting registered means that the nurse surrenders the right of nomination, the hope of being elected, the privilege of voting for the election of her friends in the nursing councils of the near future.

